# Suspicious minds: cinematic depiction of distrust during epidemic disease outbreaks

**DOI:** 10.1136/medhum-2020-011871

**Published:** 2020-05-28

**Authors:** Qijun Han, Daniel R Curtis

**Affiliations:** 1 Foreign Studies, Nanjing University of Science and Technology, Nanjing, Jiangsu, China; 2 History, Erasmus University Rotterdam Erasmus School of History, Culture and Communication, Rotterdam, The Netherlands

**Keywords:** film, infectious diseases, public health, popular media, science communication

## Abstract

One key factor that appears to be crucial in the rejection of quarantines, isolation and other social controls during epidemic outbreaks is trust—or rather distrust. Much like news reporting and social media, popular culture such as fictional novels, television shows and films can influence people’s trust, especially given that the information provided about an epidemic disease is sometimes seen as grounded in ‘scientific fact’ by societies. As well as providing information on the ‘correct science’ behind disease transmission, spread and illness in films and literature, popular culture can also inform societies about how to feel and how to react during epidemics—that is to say create some expectations about the kinds of societal responses that could potentially occur. In this article we closely analyse three films that centre around epidemic diseases—*Contagion* (Steven Soderbergh, 2011), *Blindness* (Fernando Meirelles, 2008) and *The Painted Veil* (John Curran, 2006)—in order to highlight three categories of distrust that have recently been identified and conceptualised in broader discussions regarding trust and health: institutional, social and interpersonal. These films raise two key issues about trust and social responses during epidemics. First, while certain aspects of trust are badly diminished during epidemic disease outbreaks, epidemics can also interact with pre-existing structural inequalities within society—based on race, gender or wealth—to create mixed outcomes of discord, prejudice and fear that coexist with new forms of cohesion. Second, the breakdown in trust seen at certain levels during epidemics, such as at the institutional level between communities and authorities or elites, might be mediated or negotiated, perhaps even compensated for, by heightened solidity of trust at the social level, within or between communities.

Epidemics are often seen as processes that disrupt the normal rhythms and functioning of daily life for societies. One of the characteristics of this disruption that scholars from a variety of social science and humanities disciplines have focused on over the years is the Foucauldian notion that forces from above—elites or authorities—have throughout history used epidemic disease outbreaks as a ‘tool’ for implementing oppressive social regulations and restrictions.1 In 19th-century India, for example, the classic work of David Arnold once suggested that major epidemic diseases such as smallpox, plague and cholera were instrumentalised by the colonial authorities to gain control over Indian lives and bodies.2 However, in more recent times, scholars have also drawn explicit attention to the idea that societies throughout history have not always accepted these impositions from above passively or without dissent or disorder. Furthermore, this dissent or disorder has sometimes passed under the radar of scholars focusing on the written record and ‘texts’: a view from within ‘Performance Studies’ by Dwight Conquergood suggests that the ‘oppressed everywhere must watch their backs, cover their tracks, suck up their feelings, and veil their meanings…subversive meanings and utopian yearnings can be sheltered and shielded from surveillance’.3

Implementation of quarantines and isolation has often been contested grounds, communities have disregarded regulations that infringed on social networking, and attempts to intervene or depart from customary practices such as burials or funerals have often invoked resentment and furious anger. During the cholera outbreaks in the aftermath of the 2010 earthquake in Haiti, ‘outside interests’ such as United Nations workers were blamed as the source or the spreaders of the disease. Outbreaks of Ebola in various parts of Western Africa from 2014 onwards had led to difficulties in bridging the gap between the interventions from outside authorities and afflicted local communities. Indeed, people working to stop the disease from spreading, treat the infected or simply provide information have been targeted—only in November 2019 was a Congolese journalist killed in his home for reporting on the virus,4 while later that month the WHO and other aid groups began to evacuate workers in the context of a violent siege that led to the death of a vaccination worker alongside others.5

One key factor that appears to be crucial in leading to the rejection of perceived impositions from above or outside the society is trust—or rather distrust.6 Put simply, communities that are afflicted by an epidemic outbreak are not always convinced of the course of action presented to them by authorities or elites, and this distrust likely has its roots in a variety of factors—communication failures, lack of cultural sensitivity on the part of outside agencies, pre-existing unstable and strained relations between local communities, legacies of colonialism, failures of local governments, and a lack of understanding of the economic implications that decisions such as quarantines can have, particularly for the poor and vulnerable.7 So, for example, recent survey evidence taken from the 2014–2015 Ebola outbreak in Liberia showed that respondents who expressed low trust in the government were much less likely to take protective precautions in their homes, or to abide by government-mandated social distancing mechanisms designed to contain the spread of the virus.8 Other work using similar survey methods during an Ebola outbreak found that trust was more likely to be fostered when responders—health workers, clinicians, governments officials and drivers—used more open, transparent and reflexive communication methods.9

Accordingly, communication is important, and in particular the media play a significant part in establishing trust during or in anticipation of epidemics. So, for example, it has been noted very recently how the media has perpetuated several misguided anxieties over how people can be infected with Ebola and how the disease spreads,10 and this same issue has been magnified even further in previous media representations of the transmission of AIDS-HIV, creating ill-conceived views on disease transmission, moralising overperceived ‘risky’ behaviour patterns, and stereotyped associations of the disease with particular people down the lines of race, sexuality or socioeconomic status.11 This has been framed by some as a case of the media acting as a source of ‘moral panic’,12 perhaps best assessed in accordance with Ulrich Beck’s view that a characteristic of modernity is an increased awareness of societies’ own potential for self-destruction.13

Much like news reporting and social media, other forms of popular culture such as fictional novels, television shows and films can also influence people’s trust, especially given that the information provided about an epidemic disease is sometimes seen as grounded in ‘scientific fact’ by societies, even when that is not necessarily the intention of the producer, director, writer, artist or creator.14 Audiences may be more inclined to take on this information given that educational messages are often reinforced when accompanied by emotive stories.15 Even highly implausible situations, such as those seen in zombie movies, are said to have significant public health implications.16 Indeed, answers given by the public to a Twitter forum set up by the Centers for Disease Control and Prevention (CDC) in 2011 revealed the strong link between zombies and public health awareness, bringing into popular view issues connected to contagion, mental health, ethics of disease management and bioterrorism vulnerability.17 However, as well as providing information on the ‘correct science’ behind disease transmission, spread and illness in films and literature, popular culture can also inform societies about how to feel and how to react during epidemics—that is to say create some expectations about the kinds of societal responses that could potentially occur.18 Indeed, in recent times scholars have suggested that media and popular culture have worked together to create an ‘outbreak narrative’, where the spread of an epidemic moves in one direction from ‘marginalized, deviant or underdeveloped groups’ to ‘native, mainstream, or developed society’, and accordingly play on common stereotypes connected to concepts of othering.19

The narratives and characters seen in films can provide important insights into the different dimensions of trust and breakdowns in trust that occur during or in anticipation of an epidemic disease outbreak. Indeed, by shifting between the macro-scale of humanity or society to the micro-scale of individual protagonists and relatable characters—taking in a wide range of social and demographic groups—films can, at times, do an excellent job of demonstrating how epidemic disease responses are shaped by pre-existing conditions and structures within society itself (in many contexts around the world trust in information today is already very fragile), including hierarchical and horizontal relationships between people.20

In this article we focus on a close analysis of three films that centre around epidemic diseases—*Contagion* (Steven Soderbergh, 2011), *Blindness* (Fernando Meirelles, 2008) and *The Painted Veil* (John Curran, 2006)—in order to highlight three categories of distrust that have recently been identified and conceptualised in broader discussions regarding trust and health. The reason for zooming in on these three films is that they first present epidemics in a realistic or semirealistic way, rather than overly fantastical or implausible, and have been widely watched, with cumulatively grossing worldwide figures of $135 458 097, $19 844 979 and $26 522 838, respectively.21 We categorise trust into three dimensions: (1) institutional trust, (2) social trust and (3) interpersonal trust, informed by pre-existing work on trust in public health contexts.22 First, by institutional trust we focus on societies’ hierarchical relationships developed with perceived or real elites and authorities, which comes down to people’s trust placed in medical and ‘expert’ information and trust placed in governmental authorities. Second, by social trust we focus on the relationships developed between ordinary citizens themselves as members of communities, and furthermore trust between members of communities or ‘insiders’ and those seen to be ‘outsiders’ to the communities in question. Third, by interpersonal trust we focus on the micro-scale of individual relationships between people within households, families and friendship networks. Institutional trust, therefore, refers to vertical relationships between those with unequal source of power, whereas the other two forms of trust refer to horizontal relationships—although still not always equal, it should be noted. Interpersonal trust remains largely between kin members, friends or close neighbours, whereas social trust refers more to civic society, collective institutions and broader networks beyond the family or friends.

Overall, in this paper our analysis establishes and supports two arguments. First, while certain aspects of trust are badly diminished during epidemic disease outbreaks, epidemics can also interact with pre-existing structural inequalities within society—based on race, gender or wealth—to create mixed outcomes of discord, prejudice and fear that coexist with new forms of cohesion. Historical research in recent times has argued down similar lines, emphasising that social responses to epidemics going back into the past have also included empathy and compassion,23 and this is a message often lost beyond the spectacular stories of oppression, panic and scapegoating. Indeed, the recent global COVID-19 pandemic has brought out a diversity of social responses from a wide spectrum—on the one hand a plethora of evidence pointing to incidents connected to discrimination, prejudice or xenophobia,24 and yet on the other hand likely outnumbered by examples of online and social media messages of solidarity,(Anon, 2020c) local voluntary work to tend to the vulnerable, public expressions of gratitude towards ‘key workers’, charitable donations of time and resources, and expressions of collective solidarity in the form of artistic expression and song.25 Second, the breakdown in trust seen at certain levels during epidemics, such as at the institutional level between communities and authorities or elites, might be mediated or negotiated, perhaps even compensated for, by heightened solidity of trust at the social level, within or between communities.

## *Blindness* (Fernando Meirelles, 2008)

*Blindness* is a 2008 English-language film adaptation of a Portuguese novel ‘*Ensaio sobre a Cegueira*’ (José Saramago, 1995), about a society dealing with a case of epidemic blindness. At the beginning of the film, a Japanese man (Yūsuke Iseya) suddenly goes blind in his car at an intersection, his field of vision turning white. Although initially receiving little aid, he is eventually helped by someone who drives him home, only to later steal his car. When his wife comes back, he is taken to an ophthalmologist (Mark Ruffalo), who cannot determine the exact causes of this problem. Directly the next day, the doctor himself also goes blind, and given that we see around the city many other citizens also going blind we learn that this is a contagious disease. As the numbers of afflicted rise, this becomes a serious epidemic, and one where the government responds by enforcing isolation for the blind into an old derelict abandoned asylum. We also learn that not everybody contracts the disease—the wife of the ophthalmologist (Julianne Moore) retains her vision, but ends up in the facility to protect her husband, keeping her sight a secret from everyone.

In the asylum, a number of other characters also enter—the Japanese couple, the thief, a sex worker, a young boy, an old man with an eye patch and a transistor radio, and many other patients of the doctor. By this time, the epidemic has become a global pandemic, and now given the name the ‘white sickness’. Conditions deteriorate in the camp due to lack of facilities and lack of supporting staff, leading to declines in hygiene and health. Lack of resources and food, however, ends up escalating into new lines of conflict—soldiers who guard the facility become hostile, but also resentment between the wards themselves. One ward with access to weaponry ends up threatening the others into submission by taking control of the food supplies, demanding first the valuable goods from other wards, and then coercing sex from women. During a rape, one woman is killed, and this leads to a full-on conflict. Out of the chaos of a burning building, a number of the infected manage to escape, aided by the fact that the guards had already abandoned their posts, only to find that outside society was in a similar state of collapse. On securing food, the sighted wife of the doctor invites their family to their apartment, where they attempt to establish a safe mutually supportive residence. At the end some of the characters begin to regain their sight—some celebrate, although for others this is seen as a mixed blessing.

In *Blindness*, we see the three kinds of distrust played out across the course of the film. The breakdown in trust in the decisions and actions of elites and authorities is clear, establishing itself as a form of institutional distrust. It is perhaps not an accident that the film-maker decided to place the blind in isolation at a defunct mental asylum, an action that happens both quickly and without any dialogue between officials and the infected. Parallels are drawn between the moral treatment of those deemed to be ‘insane’, as already depicted in classic cinema such as *One Flew Over the Cuckoo’s Nest* (Milos Forman, 1975), where the asylum becomes a context for the oppressive and repressive imposition of rationalised order.26 In *Blindness*, the infected are forcibly removed into isolation and then placed under camera supervision and physically watched by guards with weapons at postings high above the facility. We also see the eradication of trust in medical authority in the film, as one of the main protagonists, the male ophthalmologist, begins life in the isolation facility as his ward’s ‘official representative’, but over time as conditions worsen, the other infected people lose confidence in him, and he becomes usurped by others, including his own wife. This later is manifested further in a case of strong interpersonal distrust as the ophthalmologist then goes on to have sex with the woman presented as a sex worker, a betrayal of strong significance given that his wife who is not infected with blindness had spent much of the film voluntarily guiding and supporting him and others in the facility.

Inside the facility itself, different forms of social distrust emerge across the course of the film, particularly as more people are placed into isolation and the wards become fuller. It is not clear whether the choice of a Japanese man as the first person to be infected with the blindness is a subconscious form of othering committed by the director, or a conscious and deliberate critique of this phenomenon that is frequently seen during epidemics. This has a long history, as seen in the victimisation of Chinese people in Chinatowns in the USA during the Third Plague Pandemic outbreaks,27 and has manifested itself in xenophobic abuse reported towards Asians in many parts of Western Europe during the early phases of the COVID-19 outbreak.28 Whatever the reality of the director’s intentions, it is a useful entry into the issue of social distrust as the film’s starting scene. As the Japanese man is unable to drive through his sudden blindness, people are impatient and honk their horns. As he shouts “help,” nobody comes to his aid. In the end, he is aided by someone, but even this turns out to be an abuse of trust as this man attempts to steal items from the Japanese man’s house and then takes his car.

Social distrust is an issue further developed in the moralising take on the specific choice of victims of the disease. Not everyone in society is afflicted, and it appears as though this sudden contagious blindness is being presented as a punishment or corrective for perceived immoral behaviour. This kind of moral story has its roots in how previous societies have ‘explained’ outbreaks of certain diseases—for example, the providential notion that the Black Death and recurring plagues thereafter were a punishment from God for selfishness, greed or lack of piety.29 In the film itself, reference is made to this issue. Statues in the church, including a crucified Jesus, have all been blindfolded, and the eyes of saints in the stained-glass windows have been taped over. The characters in the film have differing views on its symbolism: some suggesting that it refers to a show of solidarity with those who are blind, while others point to the likelihood of a shattered faith, hinting even to a distrust in God herself. Those who become blind are presented in moralising terms: the thief ends up blind himself, and is punished further when attempting to grope a woman in the isolation facility, receiving a wound that later proves fatal. The sex worker is also presented in moralising terms, although perhaps more relating to the attitudes of others towards her. Once blind, she is abandoned by her client and left to fend for herself, until she is humiliated and thrown out of the hotel naked. These examples contrast with the situation of the wife of the ophthalmologist, whose apparent caring and selfless nature is reflected in the fact that she is one of the only characters not to go blind in the film. She continues to demonstrate those characteristics throughout the film—first tending to the immediate needs of her husband, even forgiving him of infidelity; second, working to ensure the safety and protection of others in her ward; and third, once escaped from the facility, guiding her followers to safety.

A final aspect of social distrust depicted in the film can be seen within the divisions of the isolation facility itself: in the end wards do not work together but compete with each another, as seen in the dramatic conflict played out between ward 1 and ward 3. In a desperate power struggle, certain members of ward 3 end up using the threat of violence—based on their access to weaponry—to hoard resources and food away from the other wards. This is then turned into an ultimatum whereby the men of ward 3 require coerced sex from the women of ward 1, in exchange for rations. This demand even eventually leads to the death of one of the women. The whole situation descends into a case of cruelty, abuse and extreme violence, and a final violent struggle between the two wards becomes the final context for members of ward 1 to escape from the facility entirely.

However, although *Blindness* focuses on many aspects of distrust, which in the end are manifested through various forms of hatred and violence, the film also emphasises different ways in which epidemics can serve to establish new lines of trust between certain groups and individuals, leading the way to heightened forms of social cohesion. Indeed, although on the one hand the disease itself is partially framed in a moralising narrative as a punishment for selfishness and greed, on the other hand the pre-existing inequalities and injustices serve as a background context which is reshaped during the ‘abnormal’ conditions of the epidemic.

The film achieves that very clearly by zooming in on the particular changing status of certain individual characters: bringing out the worst, but also the best in people. The woman with the dark glasses is a ‘part-time’ sex worker, struck blind while with a client. Although entering the facility cold and condescending, the woman takes on new roles and responsibilities which reflect a more complex and nuanced identity, beyond the mere categorisation as a sex worker. Eventually, this woman becomes a protective caregiver for a young boy, whose mother was not found in the isolation facility, and she strikes up a loving bond with an older man with a black eye patch (Danny Glover). This man urges us to reflect on our prejudices towards seeing this woman as one thing: reassuring her “I know the part inside of you with no name.” As the last man to join the facility, he also sees his fortunes change in the context of the ongoing epidemic. On escaping isolation and once in the safe confines of the ophthalmologist’s house, the man reflects on his status both preblindness and postblindness, noting that prior to the disease his status was defined by his ‘otherness’—perhaps down the lines of racial discrimination, perhaps through poverty or perhaps simply through his eye patch. For this man then, the disease had an egalitarian effect—for the first time simply accepted as a person like any other. The same story can also be found within the facility itself in the fortunes of the man who has been blind since birth, although this time not presented in favourable terms. As an experienced blind person, he is the only one in the ward who can read and write braille, can use a walking stick, and has a better command of his other senses. In the end, this man uses this reversal of fortune for his own individual gain, however, becoming second in command to the man with the gun in ward 3, in the process making money and exploiting the women of ward 1.

While on the one hand the contrast between wards 1 and 3 points to inevitable conflict, elements of cohesion also come to the surface. As ward 3 becomes an inhospitable environment, ward 1 takes on the features of a close-knit family. Interestingly, we never learn any of the names of anyone in the film, and that serves to emphasise the breakdown in social hierarchy seen in the isolation facility. Much of the solidarity seen in the film is also presented down gender lines, with an inequitable proportion of burdens and responsibilities falling on women. As noted above, one of the most traumatic events taking place in isolation is the rape of a number of women from ward 1 by men from ward 3. The film’s presentation of this kind of situation is perhaps understandable given that a wide range of scholarly and charitable literature now emphasises that terrible hazards and shocks—epidemics, famines, floods, earthquakes and the like—tend to afflict women and girls to a much more severe degree than men.30 In the beginning, the blind women of ward 1 are subjected to the harrowing experiences of sexual abuse by men from ward 3, sacrificing themselves to secure food and resources for the rest of ward 1. In the end, however, under the leadership of the doctor’s wife, the women work together with collective solidarity to instigate a successful overthrowal of the violent and despotic group of male bandits, and lead the rest of the ward to escape the confines of the institution.

## *The Painted Veil* (John Curran, 2006)

*The Painted Veil* is a 2006 American drama film that is based on a novel from 1925 written by English playwright, W Somerset Maugham, and is the third film adaptation following the initial 1934 version starring Greta Garbo and Herbert Marshall and a 1957 version (called *The Seventh Sin*) featuring Bill Travers and Eleanor Parker. The film takes place in the 1920s and follows the attempts of a British bacteriologist to help bring a cholera outbreak under control in a rural and remote area of China. The story begins in London, where the bacteriologist Walter Fane (Edward Norton) proposes marriage to a vain socialite Kitty Garstin (Naomi Watts). Eventually they end up in Shanghai, where Walter is working in a government laboratory studying infectious diseases.

From the very beginning, signs of strain can be seen in their relationship: Walter entirely consumed by his work and Kitty more interested in the social life of the British high society in Shanghai. Ultimately, this leads to an affair between Kitty and Charles Townsend (Liev Schreiber), a married British vice consul, and as a punishment Walter forces his wife to accompany him on a dangerous and uncomfortable journey to the remote village where a cholera outbreak had taken hold. Living conditions are squalid, and Kitty finds herself isolated, unable to talk to Walter, with only the company of a Chinese housekeeper (*amah*) and soldier assigned to guard her, and a British deputy commissioner, Waddington (Toby Jones), as a next-door neighbour. To escape her boredom, Kitty volunteers at the local orphanage run by a group of Catholic missionaries (led by Diana Rigg), and it is in this context that she begins to see her husband in a new light, recognising his selfless and caring side. As the epidemic becomes more severe, the relationship between the two solidifies.

Much of the story addresses the issue of the cholera outbreak. Walter tries to implement new public health controls, which are initially rejected and resisted by the local population, but eventually are accepted. Just as the situation is under control, however, displaced refugees from other cholera-stricken communities come into the village, creating new sources of contagion. Walter has no option but to set up a quarantine camp outside the settlement, but in the process becomes infected and dies a grisly and painful death. Kitty, by this time pregnant, leaves China. The film ends some 5 years later in London as Kitty, accompanied by her young son Walter, bumps into Charles by chance and rejects his advances.

Interpersonal distrust has a substantial role in *The Painted Veil*—just as in *Blindness* manifesting itself in an extramarital affair, and in turn only exacerbating distrust further. Partially this is explained by the selfish behaviour of Kitty and her superficial desires for accepting Walter’s proposal (she just wanted to get as far away as possible from the oppressive control of her mother), but also as a result of Walter’s personal flaws—his coldness, poor communication and lack of empathy—and the loneliness derived from an unfamiliar, and perhaps even directly hostile, new cultural environment. However, it could also be said that most of the interpersonal distrust in the film serves as a backdrop to the incipient stages of the epidemic, but as the cholera worsens the relationship between the two protagonists strengthens. Another form of interpersonal distrust is seen in the mysterious and seemingly ambiguous position of British neighbour, Waddington. The viewer at first is unsure to what extent the relationship between Waddington and a young Chinese woman, Wan Xi (Lü Yan), is exploitative or not—Wan Xi is presented in a stereotypically Orientalised image as exotic and mysterious, but at the same time submissively returning to Waddington even after he has sent her away a number of times. This ambiguity can be seen in a later interaction between Kitty and Waddington: Kitty is about to hand over a personal letter to Waddington—in the hope that he can help her—but decides not to do so by showing second thoughts about whether he could be trusted.

Social and institutional distrust are difficult to untangle from one another in *The Painted Veil*, largely because the lines between who is the elite or authority and who is not are rather blurred. Walter brings with him ‘modern’ Western knowledge of biomedicine, but also has to operate within an arena where he is subject to the threat of violence, ceding authority to a Chinese army general in the broader context of nationalist uprisings and anti-Western sentiment against quasi-colonial occupation. The context of nationalist sentiment—already stirred up in the student protests over the perceived injustices of the Treaty of Versailles in 1919 (the May Fourth Movement)—was further agitated by the anti-imperialist May Thirtieth Movement in response to the British Shanghai Municipal Police opening fire on Chinese protesters. In that kind of political background, we can understand the villagers’ deep-rooted distrust in Walter, not only as an outsider, but as someone to them representing British colonial presence within China. Local agitation is perhaps only exacerbated by other forms of ‘outsider intrusion’, such as the central presence of Catholic missionaries within the village.

However, while anti-imperialistic sentiment may provide the background for distrust, much is also related to Walter’s own personal characteristics—as a bacteriologist used to working in a laboratory rather than a field doctor, he lacks some of the ‘softer’ skills required in dealing with sick people and forging useful relationships and networks. Accordingly, important figures such as the army general, Colonel Yu (Anthony Wong), have an initially frosty relationship with Walter. In one telling scene, Colonel Yu tells Walter that he thinks “China belongs to Chinese people, but the rest of the world seems to disagree,” whereas Walter is unreceptive to the political context, simply replying “That’s got nothing to do with me. I didn’t come here with a gun, you know. I came here with a microscope.” However, it should also be noted that the ‘outsider’ concept within the film is multilayered, since a level of distrust also exists between Colonel Yu himself and some of the ordinary inhabitants of the village.

It is clear that the initial solutions put forward by Walter—informed by a completely different framework of disease management—were also not easily accepted by the local village population. Indeed, the first time we see the rising discontent of villagers is when Walter suggests moving bodies from burial site close to the river to avoid contaminating the water, something which stands in direct conflict with the customary practices of placing the dead closer to the river to move on more swiftly to the afterlife. This anger was further exacerbated by Walter’s additional suggestion to remove all corpses and bury them as soon as possible—this time in conflict with the customary expectation of allowing a number of days to elapse with the bodies laid out in the house before burial. In one dramatic scene, we see the anguish etched on the faces of the frantic villagers, as soldiers march into the households and carry off the corpses. On the one hand we may be slightly suspicious of this narrative from a Western director, perhaps subscribing to a kind of Orientalist imagination about the ‘backwards’ or ‘primitive’ practices that might have occurred in a rural village in early 20th-century China, but at the same time medical history does seem to at least verify some of this narrative. Indeed, in the 1920s and 1930s in rural China, ‘outsider’ and elite medical reformers’ lack of attention paid to internal village politics and power dynamics limited the overall effectiveness of public health prevention and education, as local populations held on to the same routines.31

Although *The Painted Veil* deals with many cases of distrust on all three levels—interpersonal, social and institutional—we could also see that the film also provides a clear narrative in which trust becomes fully re-established. This occurs across two dimensions: first in the personal relationship between Walter and Kitty themselves, and second in Walter’s interactions with the local community that facilitates his public health interventions. In the case of Walter and Kitty, as already noted, we find that the worsening of the cholera crisis within the village corresponds temporarily with the strengthening of their marriage. As Walter loses himself ever further in his work, Kitty becomes isolated to an even greater degree, forcing her to carve out a new role for herself within the orphanage run by French nuns. It is only in this context that the couple see a different hidden side to each other, and become reconnected via their emotional attachment to issues taking place within the village itself. In doing so, the cholera crisis also serves as a context for Kitty to begin to establish her own trust of the community itself—perhaps even to a greater degree than Walter. At the beginning, Kitty is lonely and uncomfortable, infuriated by her interactions with the *amah* (Gesang Meiduo), caught staring at her, and the overbearing protection of bodyguard, Sung Ching (Li Feng), told to stop following her around. Yet as the film progresses, Kitty is found readily accepting the services of Sung Ching, and in one scene continues to eat the uncooked salad of the housekeeper, despite the protestations of Walter.

Although Walter encounters resistance to his ideas through widespread distrust in the local community, he is eventually able to win the confidence and cooperation of the people by better establishing a more reciprocal relationship within local power hierarchies. At first, the army general attached to the village shows hostility to the presence of an ‘outside’ authority dictating terms, yet on establishing a dialogue the relationship between the two improves, and in one case Colonel Yu saves Walter and Kitty from the advances of a malicious group of locals by firing a gun. Colonel Yu and Walter begin to establish trust on the basis of a shared respect for their respective professionalism, but also common language and open-mindedness, and at the end of the film the general is found weeping on witnessing Walter’s death.

The establishment of trust between Walter and the community, however, is only confirmed once Walter gets the approval from a local feudal warlord for his ideas of establishing an aqueduct of fresh running water across large distances. The film is unclear about whether the eventual cooperation of the villagers stemmed from being convinced by the approval of the local lord or stemmed from overall fear and submission to power, but what is apparent is that his support remained vital. Intriguingly, this warlord himself remained entirely unconvinced until the army general stepped in as a form of conversational buffer, using both his local authority and his cultural position to know exactly what to say and how to frame things. After Walter insults the warlord in English—“Tell him that’s the most ridiculous suit that I’ve ever seen”—Colonel Yu turns to the warlord and remarks: “This Doctor respects you greatly, and you are right. It is quite a mess, this epidemic. But my superior said if your men cannot control it, then our army will be happy to come out here and help you.” The combination of praise, respect and a thinly veiled threat from the army general helps Walter negotiate the difficulties of local political networks and power hierarchies.

## *Contagion* (Steven Soderbergh, 2011)

Soderbergh’s *Contagion* was produced in 2011, appearing in the aftermath of a number of serious epidemic outbreaks including severe acute respiratory syndrome and various forms of influenza (H1N1/H5N1). The film centres on the unfolding of a global pandemic, which we later find out is a mutated virus derived of genetic material from bats and pigs (given the name meningoencephalitis virus 1 or MEV-1), and by the time a vaccine has been developed the seriousness of this situation is seen in a projected infection rate of 8.3% and a mortality rate of around 25%–30%. The film begins on ‘day 2’ of the outbreak in Hong Kong and then proceeds to show the spread of the infection across many parts of the world across hundreds of days. At the end of the film, we are taken back to ‘day 1’, where we are shown the origins of the disease: the disruption of the natural habitat of a bat by human construction pushes the bat to fly over a pigpen, in the process dropping a piece of banana. A pig consumes the banana, which is then sold by farmers to a chef in a Macau casino. The failure to wash his hands while handling the pig carcass passes the virus to an American woman, Beth (Gwyneth Paltrow), travelling on business, after he shakes her hand.

Across the course of the film, the viewer is taken from various locations—Chicago and Minneapolis in the USA, where Beth travelled back to from Hong Kong, as well as scenes of the infection unfolding in London, Hong Kong, Macau, and other parts of China such as Guangdong. As a global outbreak we are taken through the responses of various kinds of institutions and actors—governments, disease management authorities (the CDC), global health authorities (the WHO), media outlets, pharmaceutical companies, hospitals and many more, but the macro-level analysis of a global pandemic is also broken down into the micro-level through the numerous overlapping stories of the individual protagonists, from elites and authorities to ‘ordinary’ citizens. Although we see many of these characters in their ‘official role’ as doctors, epidemiologists, scientists and the like, we also come to see that these same characters have personal lives beyond their jobs—emotional connections with others that also dictate the nature of their response during the epidemic, and perhaps lead them to deviate from established protocols required by formal institutions.

Overall, the virus presented in the film is not just the cause of a global pandemic, but is also a metaphor for fear and distrust, often heightened through media messages, and further exacerbated down socioeconomic, cultural and racial lines. Just as in *Blindness* and *The Painted Veil*, an extramarital affair is instrumentalised in the film as a way of demonstrating a major case of interpersonal distrust. Mitch Emhoff (Matt Damon) finds out that his deceased wife, Beth, was unfaithful with an ex named John Neal, which accordingly leads to the spread of the disease in Chicago.

Quite frequently, strong levels of distrust down various lines turn into levels of mistrust: that is people believing in spurious forms of information. This can be seen clearly in the institutional distrust in authorities—both medical and governmental—which leads to mistrust in online sources of information peddling pseudoscience. Throughout the film, ‘expert’ opinion gets sidelined in favour of blogs, which conspiracy theorists such as Alan Krumwiede (Jude Law) exploit to spread unsubstantiated allegations about the disease, such as that it was deliberately engineered. As we go further into the worst phases of the epidemic, as well as discrediting other authorities such as the CDC and the WHO, Krumwiede pretends to be sick so he can ‘cure’ himself with a homeopathic remedy, forsythia, and presents this to millions of followers online—a financially lucrative move given that he stands to make money from promoting the same remedy. The actions of authorities in charge of distributing resources are also consistently brought into question. In two cases, ordinary people are waiting in anticipation of receiving drugs and food, but the sudden rationing of these resources among a distrusting population inevitably leads to uproar and violent confrontation.32 The kidnap of WHO epidemiologist (Marion Cotillard) by a Chinese official, Sun Feng (Chin Han), is done with the realisation that the distribution of eventual vaccines was not likely to be an equitable process.

Social distrust is also present in the film in various forms—understandable given the contagiousness and lethality of the virus. Stereotypical social breakdown scenes are found in certain places: the violent looting of banks, stores and people’s unprotected homes, dysfunction in police responses, and the build-up of uncollected trash around various cities. On realisation of these dangers, one of the film’s major protagonists, Mitch Emhoff, acquires a shotgun for himself as a form of protection. *Contagion* also zooms in on forms of exoticism and othering often seen during epidemic responses, although like with *Blindness* and *The Painted Veil* it is difficult to ascertain whether this is a conscious critique or a subconscious employment of the same flawed approaches. It is likely no coincidence that the roots of the disease are seen in Macau and Hong Kong, and the explanatory scene of how the disease unfolded could be interpreted as a negative view of Chinese development encroaching on natural ecosystems, Chinese pig farming practices and Chinese culinary hygiene. The preoccupation with filth is seen when Li Fai (Chui Tien You) is seen stumbling through the streets in a feverous state, with his vision zooming in on crowded and chaotic seafood markets. Despite this, most of the proactive ‘life-saving’ decisions are made by Westerners, and the only response we see in East Asia is the eventual kidnap and bargaining of a white epidemiologist by a Chinese government official, Sun Feng. The same woman seemingly returns to the village where she was kidnapped as the potential saviour and ‘white knight’.

Although at the heart of *Contagion* is a clear story of distrust along all three lines—institutional, social and interpersonal—the film also shows elements of solidarity and compassion between some of the characters. Frequently this is demonstrated in the courageous and skilled work of many of the public health professionals in dealing directly with the disease, often going beyond the ‘call of duty’ to help others at great risk to their own lives. We see Dr Ally Hextall (Jennifer Ehle) working consistently in a secured lab to try and isolate the virus and develop a serum. In the end, she risks her own life by testing the vaccine on herself. The same could be said of the WHO epidemiologist sent to Hong Kong to find the origins of the MEV-1 pathogen. Although effectively kidnapped by Sun Feng, and used as leverage to obtain vaccines for his family’s village, Dr Orantes not only is found educating the local children in one scene, but on realising that the village were given placebos instead of real vaccines voluntarily goes back there to help. Similar types of actions were seen in the CDC workers. Dr Erin Mears (Kate Winslet), an epidemic intelligence officer, begins investigating the situation in Minneapolis but in the process is infected herself. In her final act, she is seen passing a coat to another sick person for warmth, just before she dies. She is then zipped up and thrown into a mass burial site—a cold and brutal scene that neither glorifies Dr Mears as a hero nor reflects too long with sentiment or tragedy. The female burden and self-sacrifice again appear as themes: mirroring the women-led escape from the institution in *Blindness*, and Kitty’s transformation into principal caregiver in *The Painted Veil*. Indeed, Dr Mears’ self-sacrifice is brought into further focus by being juxtaposed against a decision to divert a flight, originally scheduled to pick her up, to instead collect an ‘important’ politician.

The CDC director (Laurence Fishburne) is also seen to show compassion in the face of difficult circumstances—at the end of the film eschewing his right to an early vaccine and giving it to a small boy from a poorer family. Furthermore, Dr Cheever takes direct responsibility for perceived mistakes: accepting with humility the investigation into his behaviour, after he had told his fiancée to evacuate before announcing it publicly. It must also be noted, however, that these compassionate responses often come out of breaking established rules and protocols from their superiors or institutions. Dr Cheever himself explicitly states later in the film that he would do the same again—that is break the official rules—if given the choice. Dr Hextall continues to work on the vaccine despite instructions for her to stop. Accordingly, the film shows an uneasy coexistence between decisions made out of compassion for others, but usually only enabled by disregard for official practices—a disregard to some extent created by prevailing levels of institutional distrust.

Another aspect of the film that concerns the re-establishment of trust is the continual attempts to maintain close personal networks with others and ordinary practices—something that the epidemic problematises. Indeed, this is an issue recently brought into focus during the early phases of the COVID-19 outbreak in China, where a WeChat diary from a quarantined Wuhan resident noted that ‘it is not easy to build trust and bonds under a lockdown’ but at the same time ‘social participation is an important need’.33 On a number of occasions we see the two teenagers, Jory Emhoff (Anna Jacoby-Heron) and Andrew (Brian O’Donnell), attempting to meet each other and socialise, despite the best efforts of Jory’s father to separate them. The need for the continuance of ordinary life is seen at the end of the film, where the father puts on a special ‘Prom Night’ dance for the pair at their own house—in light of the isolation conditions—and informing his daughter “It’s gonna start getting normal again.” Earlier in the film, the same father was left distraught when he was told by the undertaker that he could not bury his wife and son at his cemetery, despite the fact that he already had a family plot.

## Conclusion

The three films chosen for analysis in this paper—*Blindness*, *The Painted Veil* and *Contagion*—each reflects on the capacity of different societies to exhibit levels of distrust during and in anticipation of epidemic disease outbreaks. These elements of distrust can be classified as institutional, where communities lose confidence in the actions and decisions of elites and authorities; social, where communities begin to distrust each other, or more frequently, ‘outsiders’ to these communities; and interpersonal, where individual relationships within families, households, friends and neighbours begin to break down. A recurring feature within these three films is the use of affairs between the main protagonists to demonstrate suspicion and distrust at the very micro-level—an approach that the medium of films and television can perform very well given their focus on individual characters. Another feature recurring within the films is a form of othering—even if it is subconscious rather than a deliberate critique—by exoticising the threat of disease transmission and spread. In *The Painted Veil*, this is turned around in the opposite direction, as the ‘exotic’—the ‘timeless rural Chinese village as the nexus of infection—is ‘saved’ by a selfless Western bacteriologist risking his own life, despite their apparent ‘incapacity’ or ‘unwillingness’ to understand the health risks presented to them. These issues, firmly placed in mainstream cinema, are of obvious relevance to us when we consider (1) the effectiveness of popular culture in presenting public health issues to a broader ‘lay’ audience, and (2) the recent portrayal of the origins and spread of COVID-19 as almost a ‘moral’ issue relating to economic practices and social life in China. It is also important given the feeling recently expressed during this very same outbreak that ordinary lives of citizens, especially the poor and marginalised, often get pushed into the background in favour of ‘macroscopic’ elements of disease management.34

These kinds of films also provide unexpected sources of nuance to how trust and distrust might be forged during epidemic outbreaks. First, in each of the films, it is emphasised how suspicion and prejudice are not features which ‘suddenly’ emerge out of entirely new conditions created by fear of the pathogen. In fact, suspicion and prejudice are often ingrained features of societies before the appearance of epidemics, and informed by structural inequalities based on race, gender and socioeconomic status. Epidemics may simply bring these characteristics to the surface, holding them up to the light. Second, and on a related note, the three films of analysis also suggest that while epidemics will often exacerbate and pull apart pre-existing inequalities, this might not be a universal rule. Frequently, we find situations where both distrust and cohesion can coexist simultaneously, with the lines between the two more blurred than what appears on the surface. Indeed, this point can be reinforced when approaching these films through the three levels of breakdown in trust, where institutional distrust—often by communities towards elites or authorities—is sometimes mediated or negotiated, perhaps even compensated for, by reinforced solidarity of trust at the social level, within or between communities.

## Notes

1. On instrumentalising sickness, Michel Foucault (1995), *Discipline and Punish: The Birth of the Prison* (New York: Vintage Books), 195–200.

2. David Arnold (1993), *Colonizing the Body: State Medicine and Epidemic Disease in Nineteenth-Century India* (Berkeley, CA: University of California Press).

3. Dwight Conquergood (2002), “Performance Studies: Interventions and Radical Research,” *TDR/The Drama Review* 46, no. 2: 148.

4. Anon (2019b), “Ebola: Attackers Kill DR Congo Journalist Shining Light on Virus,” *BBC News*, November 3, 2019, https://www.bbc.com/news/world-africa-50283286.

5. Amy Maxmen (2019), “Ebola Responders Killed as Violence Flares,” *Nature*, November 26, 2019, https://www.nature.com/articles/d41586-019-03667-1.

6. Anon (2019a), “Building Trust Is Essential to Combat the Ebola Outbreak,” *Nature* 567, no. 7749: 433.

7. Samuel Cohn and Ruth Kutalek (2016), “Historical Parallels, Ebola Virus Disease and Cholera: Understanding Community Distrust and Social Violence with Epidemics,” *PLoS: Current Outbreaks* 8: 1, https://doi.org/10.1371/currents.outbreaks.aa1f2b60e8d43939b43fbd93e1a63a94.

8. Robert A. Blair, Benjamin S. Morse, and Lily L. Tsai (2017), “Public Health and Public Trust: Survey Evidence from the Ebola Virus Disease Epidemic in Liberia,” *Social Science & Medicine* 172: 89–97. Also emphasising sociopolitical arrangements, Annie Wilkinson and James Fairhead (2017), “Comparison of Social Resistance to Ebola Response in Sierra Leone and Guinea Suggests Explanations Lie in Political Configurations Not Culture,” *Critical Public Health* 27, no. 1: 14–27.

9. Molly J. Ryan, Tamara Giles-Vernick, and Janice E. Graham (2019), “Technologies of Trust in Epidemic Response: Openness, Reflexivity and Accountability during the 2014-2016 Ebola Outbreak in West Africa,” *BMJ Global Health* 4, no. 1: e001272.

10. William B Millard (2015), “Ebola Preparedness: On Avoiding Making a Scary Virus Scarier,” *Annals of Emergency Medicine* 65, no. 2: A15–20; Shannon M. Blakey et al. (2015), “Tracing ‘Fearbola’: Examining the Psychological Predictors of Anxious Responding to the Ebola Virus,” *Cognitive Therapy Research* 39: 816–25.

11. Michael S. Sinha and Wendy E. Parmet (2016), “The Perils of Panic: Ebola, HIV, and the Intersection of Global Health and Law,” *American Journal of Law and Medicine* 42, no. 2–3: 223–55; Johanna Hood (2011), *HIV/AIDS, Health and the Media in China: Imagined Immunity through Racialized Disease* (Abingdon: Routledge); Shamshad Khan (2014), “Manufacturing Consent?: Media Messages in the Mobilization Against HIV/AIDS in India and Lessons for Health Communication,” *Health Communication* 29, no. 3: 288–98.

12. Sander L Gilman (2010), “Moral Panic and Pandemics,” *The Lancet* 375, no. 9729: 1866–7; Stephen L Muzzatti (2005), “Bits of Falling Sky and Global Pandemics: Moral Panic and Severe Acute Respiratory Syndrome (SARS),” *Illness, Crisis & Loss* 13, no. 2: 117–28.

13. Ulrich Beck (1986), *Risk Society: Towards a New Modernity* (New Delhi: Sage).

14. Christopher H Foreman, (1995), “Editorial Commentary: Witchcraft Science in the Cinema of Epidemics,” *Science Communication* 17, no. 1: 3–8; Georgios Pappas et al. (2003), “Infectious Diseases in Cinema: Virus Hunters and Killer Microbes,” *Clinical Infectious Diseases* 37, no. 7: 939–42; Evie Kendal (2019), “Public Health Crises in Popular Media: How Viral Outbreak Films Affect the Public’s Health Literacy,” *Medical Humanities* Online First: 1–9, https://doi.org/10.1136/medhum-2018-011446.

15. V. Freimuth et al. (2000), “Communicating the Threat of Emerging Infections to the Public,” *Emerging Infectious Diseases* 6, no. 4: 343.

16. Brandon Brown et al. (2015), “Responsible Use of Pop Culture and Communication in the Face of Ebola Virus,” *PLoS Neglected Tropical Diseases* 9, no. 8: e0003890; Melissa Nasiruddin et al. (2013), “Zombies – A Pop Culture Resource for Public Health Awareness,” *Emerging Infectious Diseases* 19, no. 5: 809–13.

17. Nasiruddin *et al.*, “Zombies,” 812.

18. Priscilla Wald (2008), *Contagious: Cultures, Carriers, and the Outbreak Narrative* (Durham, NC: Duke University Press); Kirsten Ostherr (2005), *Cinematic Prophylaxis: Globalization and Contagion in the Discourse of World Health* (Durham, NC: Duke University Press); N Tomes (2002), “Epidemic Entertainments: Disease and Popular Culture in Early-Twentieth-Century America,” *American Literary History* 14, no. 4: 624–9.

19. On the ‘outbreak narrative’, Wald, *Contagious*. Also, on this theme, Kirsten Ostherr (2002), “Contagion and the Boundaries of the Visible: The Cinema of World Health,” *Camera Obscura: Feminism, Culture, and Media Studies* 17, no. 2: 1–40; Kendal, “Public Health Crises,” 1–9.

20. Qijun Han and Daniel R. Curtis (2020), “Social Responses to Epidemics Depicted by Cinema,” *Emerging Infectious Diseases* 26, no. 2: 389–94.

21. The Painted Veil (2016),” IMDb, accessed January 25, 2020, https://www.imdb.com/title/tt0446755/?ref_=nv_sr_srsg_1; “Blindness (2008),” IMDb, accessed January 25, 2020, https://www.imdb.com/title/tt0861689/?ref_=nv_sr_srsg_0; “Contagion (2011),” IMDb, accessed January 25, 2020, https://www.imdb.com/title/tt1598778/.

22. Julie Brownlie (2008), “Conceptualizing Trust and Health,” in *Researching Trust and Health*, eds. Julie Brownlie, Alexandra Greene, and Alexandra Howson (New York: Routledge), 20–1.

23. Samuel Kline Cohn (2018), *Epidemics: Hate and Compassion from the Plague of Athens to AIDS* (Oxford: Oxford University Press).

24. Anon (2020) “List of Incidents of Xenophobia and Racism Related to the 2019–20 Coronavirus Pandemic,” *Wikipedia*, accessed April 26, 2020, https://en.wikipedia.org/wiki/List_of_incidents_of_xenophobia_and_racism_related_to_the_2019%E2%80%9320_coronavirus_pandemic.

25. Anon (2020a), “Acts of Kindness Spread Amid COVID-19 Outbreak as UN Acts to Counter Threat,” *United Nations: COVID-19 Response*, March 27, 2020, https://www.un.org/en/coronavirus-disease-covid-19/acts-solidarity-spread-amid-covid-19-outbreak-un-continues-counter.

26. Michel Foucault (1961), *Folie et Déraison: Histoire de la folie l'âge classique* (Paris: Librairie Plon).

27. Gunther B Risse (2012), *Plague, Fear, and Politics in San Francisco’s Chinatown* (Baltimore: John Hopkins University Press); Nayan Shah (2001), *Contagious Divides: Epidemics and Race in San Francisco’s Chinatown* (Berkeley, CA: University of California Press).

28. Anon (2020b), “Coronavirus: French Asians Hit Back at Racism with ‘I’m Not a Virus’,” *BBC News*, January 29, 2020, https://www.bbc.com/news/world-europe-51294305.

29. A view often associated with contemporary observations in Florence during the Black Death: Giovanni Boccaccio (1972), *Decameron*, trans. G.H. McWilliam (Harmondsworth: Penguin Classics), 50–8.

30. Eric Neumayer and Thomas Plümper (2007), “The Gendered Nature of Natural Disasters: The Impact of Catastrophic Events on the Gender Gap in Life Expectancy, 1981–2002,” *Annals of the Association of American Geographers* 97, no. 3: 551–66. Specifically, on the gendered effects of epidemic diseases, Clare Wenham, Julia Smith, and Rosemary Morgan (2020), “COVID-19: The Gendered Impacts of the Outbreak,” *The Lancet* 395, no. 10277: 846–8; Julia Smith (2019), “Overcoming the ‘Tyranny of the Urgent’: Integrating Gender Into Disease Outbreak Preparedness and Response,” *Gender & Development* 27, no. 2: 355–69.

31. Kate Merkel-Hess (2016), “The Public Health of Village Private Life: Reform and Resistance in Early Twentieth Century Rural China,” *Journal of Social History* 49, no. 4: 881–903; Nicole Elizabeth Barnes (2018), *Intimate Communities: Wartime Healthcare and the Birth of Modern China, 1937-1945* (Berkeley, CA: University of California Press).

32. Research on food crises tends to suggest that extreme protests in the forms of physical or violent unrest occur more usually during breakdowns in ‘entitlements’—the failure of a protective authority trusted to provide ‘expected’ welfare: John Bohstedt (2016), “Food Riots and the Politics of Provisions from Early Modern Europe and China to the Food Crisis of 2008,” *The Journal of Peasant Studies* 43, no. 5: 1035–67; Louise A Tilly (1983), “Food Entitlement, Famine, and Conflict,” *Journal of Interdisciplinary History* 14, no. 2: 333–49.

33. Anon (2020d), “Coronavirus Wuhan Diary: Living Alone in a City Gone Quiet,” *BBC News*, January 30, 2020, https://www.bbc.com/news/world-asia-china-51276656.

34. Richard Horton (2020), “Offline: 2019-nCoV—A Desperate Plea,” *The Lancet* 395, no. 10222: 400.
